# Human adult stem cells derived from adipose tissue and bone marrow attenuate enteric neuropathy in the guinea-pig model of acute colitis

**DOI:** 10.1186/s13287-015-0231-x

**Published:** 2015-12-10

**Authors:** Rhian Stavely, Ainsley M. Robinson, Sarah Miller, Richard Boyd, Samy Sakkal, Kulmira Nurgali

**Affiliations:** Centre for Chronic Disease, College of Health and Biomedicine, Victoria University, Melbourne, Australia; Department of Anatomy and Developmental Biology, Monash University, Melbourne, Australia; College of Health and Biomedicine, Victoria University, Western Centre for Health Research & Education, 176 Furlong Road, St Albans, 3021 VIC Australia

**Keywords:** Enteric neuropathy, Mesenchymal stem cells, Multipotent stromal cells, Intestinal inflammation, Colitis, Myenteric neurons, Neuroprotection, Guinea-pig, Bone marrow, Adipose tissue

## Abstract

**Introduction:**

Mesenchymal stem cells (MSCs) have been identified as a viable treatment for inflammatory bowel disease (IBD). MSCs derived from bone marrow (BM-MSCs) have predominated in experimental models whereas the majority of clinical trials have used MSCs derived from adipose tissue (AT-MSCs), thus there is little consensus on the optimal tissue source. The therapeutic efficacies of these MSCs are yet to be compared in context of the underlying dysfunction of the enteric nervous system innervating the gastrointestinal tract concomitant with IBD. This study aims to characterise the *in vitro* properties of MSCs and compare their *in vivo* therapeutic potential for the treatment of enteric neuropathy associated with intestinal inflammation.

**Methods:**

BM-MSCs and AT-MSCs were validated and characterised *in vitro.* In *in vivo* experiments, guinea-pigs received either 2,4,6-trinitrobenzene-sulfonate acid (TNBS) for the induction of colitis or sham treatment by enema. MSCs were administered at a dose of 1x10^6^ cells via enema 3 hours after the induction of colitis. Colon tissues were collected 24 and 72 hours after TNBS administration to assess the level of inflammation and damage to the ENS. MSC migration to the myenteric plexus *in vivo* was elucidated by immunohistochemistry and *in vitro* using a modified Boyden chamber assay.

**Results:**

Cells exhibited multipotency and a typical surface immunophenotype for validation as bona fide MSCs. *In vitro* characterisation revealed distinct differences in growth kinetics, clonogenicity and cell morphology between MSC types. *In vivo*, BM-MSCs were comparatively more effective than AT-MSCs in attenuating leukocyte infiltration and neuronal loss in the myenteric plexus. MSCs from both sources equally ameliorated body weight loss, gross morphological damage to the colon, changes in the neurochemical coding of neuronal subpopulations and the reduction in density of extrinsic and intrinsic nerve fibres innervating the colon. MSCs from both sources migrated to the myenteric plexus in *in vivo* colitis and in an *in vitro* assay.

**Conclusions:**

These data from *in vitro* experiments suggest that AT-MSCs are ideal for cellular expansion. However, BM-MSCs were more therapeutic in the treatment of enteric neuropathy and plexitis. These characteristics should be considered when deciding on the MSC tissue source.

## Introduction

Mesenchymal stem cells (MSCs) otherwise known as multipotent stromal cells have been proposed as a potential treatment option for chronic inflammation and neurological damage [1]. These cells are identified by their multipotency as implied by the name [2]; however it is the numerous other characteristics that attract investigations into their therapeutic potential. MSCs are readily isolated from adult bone marrow or adipose tissue [3–5]. In vitro, MSCs are easily purified due to their adherence to plastic and proliferation, generating high yields of cells for treatments [6]. The immune evasive nature of MSCs may also be exploited for allogeneic and, if required, xenogeneic transplantation [7, 8]. Once administered, MSCs migrate towards sites of inflammation by chemotaxis [9]. Engrafted MSCs can then exert immunomodulatory activities and promote endogenous repair mechanisms through secretion of cytokines in addition to angiogenic and trophic factors [10–13]. These traits make MSCs ideal candidates to target both the inflammatory pathology and structural damage to the intestines of inflammatory bowel disease (IBD) patients.

Current treatments for IBD often fail to maintain periods of remission effectively throughout the prolonged course of illness due to their inefficacy or toxicity [14], thus necessitating the development of novel treatments. Clinical trials of MSC application in IBD have recognised their therapeutic efficacy, feasibility and safety. Specifically, MSCs have shown promise in the treatment of the fistulising and inflammatory luminal pathologies of Crohn’s disease [15, 16]. Despite the recruitment of patients who are refractory to conventional therapy, Forbes et al. [17] demonstrated clinical remission and endoscopic improvement in more than half of patients with Crohn’s colitis and ileocolitis. However, further studies are crucial to optimise MSC therapy for better treatment outcomes.

Investigating the facets of MSCs derived from different tissue sources may provide an opportunity to improve therapy. In experimental models and clinical trials, MSCs from either bone marrow or adipose tissue have been used. MSCs derived from bone marrow (BM-MSCs) have predominated in experimental models, whereas the majority of clinical trials have used MSCs derived from adipose tissue (AT-MSCs) [18], thus there is little consensus on the optimal tissue source of MSCs. The favoured use of AT-MSCs in the clinic is presumably owing to the availability and less invasiveness of obtaining adipose tissue [19]. Furthermore, the cell yield of AT-MSCs is predicted to be 500 fold that of BM-MSCs [20]. Nonetheless, differences in the functional efficacy of these MSCs could influence the preference of tissue source.

Studies directly comparing BM-MSCs and AT-MSCs are limited. However, MSC application in different in vivo models of various inflammatory conditions indicate that BM-MSCs exhibit functionally better immunomodulatory properties [21–24]. Conversely, AT-MSCs have been reported to be functionally better than BM-MSCs in ameliorating the clinical and pathological severity of autoimmune demyelination due to their enhanced ability to migrate to the central nervous system [25]. In intestinal inflammation, studies have largely investigated MSC protection of the mucosal integrity and polarisation of the pro-inflammatory signalling milieu [26–31]. Recently, we have demonstrated that human BM-MSCs can attenuate neuropathy in the enteric nervous system (ENS) of guinea-pigs with colitis [32]. Comparisons between BM-MSCs and AT-MSCs are yet to be elucidated in the context of inflammation-induced neurological damage concomitant with intestinal inflammation.

The colon is innervated extrinsically by parasympathetic vagus nerve fibres, sympathetic and sensory afferent fibres of the dorsal root ganglion [33]. Intrinsic innervation is governed by the enteric nervous system (ENS) which can function independently of extrinsic input and justifies its label as ‘the little brain’ [34]. The ENS consists of a network of neurons and glial cells spanning the length of the gastrointestinal tract which form the ganglia. The ganglia, containing individual neuronal subpopulations, are localised within the submucosal plexus regulating secretion and vasodilation, and the myenteric plexus coordinating muscular contraction [35]. Persistent intestinal inflammation associates with disruption to the ENS causing symptomatic gut dysfunctions [36–39]. Neuropathy and axonal damage are likely to be consequential of inflammation in the bowel [40–43]. However, in non-inflamed regions, the invasion of leukocytes to the level of the enteric plexuses, termed plexitis, may be an indicator of inflammatory relapse [44–46]. Furthermore, the neurochemical coding that defines neuronal subpopulations is altered in animal models of intestinal inflammation and IBD patients [41, 47–50]. These changes are not only responsible for the symptoms of IBD but can perpetuate further intestinal inflammation. Thus, the ENS presents as a therapeutic target for IBD. In this study we performed a comparison in the in vitro characteristics and in vivo therapeutic efficacy of BM-MSCs and AT-MSCs for the treatment of inflammation-induced neurological changes in the colon.

## Methods

### Animals

Male and female Hartley guinea-pigs weighing 140–280 g were received from the South Australian Health and Medical Research Institute (SAHMRI). All guinea-pigs were housed in a temperature-controlled environment with 12-hour day/night cycles and had ad libitum access to food and water. The average weight of guinea-pigs that underwent experimental procedures was 248 ± 5 g. All procedures were performed under approval of the Victoria University Animal Experimentation Ethics Committee (ethics number AEETH 12–012) and conducted according to the Australian National Health and Medical Research Council (NHMRC) Code of Practice for the Care and Use of Animals for Scientific Purposes.

### Cell culture and passaging

Pre-established cell lines of human MSCs (Tulane University, New Orleans, LA, USA) were derived from the bone marrow and adipose tissue of four donors. MSCs were cultured to the fourth passage for all subsequent experiments and exhibited a viability of minimum 95 % at the time of injection. Cells were plated at an initial density of 60 cells/cm^2^ and incubated in expansion medium (α-MEM supplemented with 100 U/mL penicillin/streptomycin, 1 % glutaMAX (Gibco®, Life Technologies, Melbourne, Australia) and 16.5 % foetal bovine serum (FBS; mesenchymal stem cell-qualified, Gibco®) which was replenished every 48-72 h for 10–14 days until the cells were 70-85 % confluent (maximum). MSCs were trypsinised and either reseeded for expansion or collected for in vitro experiments and in vivo treatment of guinea-pigs. All MSC cultures were incubated at 37 °C in 5 % CO_2_ throughout the study.

### Surface marker expression

MSCs were immunolabelled as previously described [51] with CD29-Alexa Fluor 488 (clone TS2/16), CD34-phycoerythrin (PE) (clone 581), CD45-PerCPCy5.5 (clone H130), CD44-Brilliant Violet 421 (clone IM7), CD73-Brilliant Violet 421 (clone AD2), and CD90-Alexa Fluor 647 (clone 5E10) (1:100) (Biolegend, San Diego, CA, USA). Data were acquired on a BD FACSCanto II flow cytometer with FACSDiva v6.1 software (BD Biosciences, Sydney, Australia). Unlabelled cells were incubated with 7-aminoactinomycin D (7-AAD) (1:20) (Life Technologies, Melbourne, Australia) for one min before acquisition to determine the viability of the cell suspensions.

### Differentiation assay

The differentiation potential of MSCs was assessed using the StemPro® Adipogenesis Differentiation Kit and StemPro® Osteogenesis Differentiation Kit according to the manufacturer’s instructions (Life Technologies). To detect adipogenesis, MSCs were fixed in 10 % neutral buffered formalin after two weeks in culture and lipid vacuoles were stained with Oil red O (Sigma-Aldrich, Sydney, Australia) in 60 % (v/v) isopropanol. Cells were then counterstained with haematoxylin. To detect osteogenesis, MSCs were fixed in 10 % neutral buffered formalin after three weeks in culture and calcium deposits were stained with 2 % (w/v) Alizarin red S (Sigma-Aldrich) in distilled water.

### Colony forming unit-fibroblast (CFU-f) assay

MSCs were seeded in 90 mm size petri dishes at low density (100 cells/dish). Expansion medium was changed every three to four days. After two weeks in culture, MSCs were fixed and stained with 0.5 % (w/v) crystal violet (Sigma-Aldrich) in methanol for 30 min before colonies containing >50 cells (CFU-f) [52] were counted under a dissection microscope.

### MSC growth kinetics and cell morphology

To assess cell proliferation, MSCs were cultured in triplicates and seeded at 60 cells/cm^2^ in 25 cm^2^ cell culture flasks containing 5 mL of expansion medium which was replaced every 48-72 h. Cells were trypsinised and counted with a haemocytometer at days 3, 7, and 14. The population doubling level (PDL) was calculated using the formula PDL = (log^2^ [final no. of cells]) - (log^2^ [initial cells seeded]) [53]. For morphological studies, MSCs were seeded at 100 cells/cm^2^ in six-well plates and analysed after 48 h. MSCs were morphologically characterised into one of two categories defined by the presence of elongated cell bodies with long thin processes (spindle) or flat bodies with irregular processes (flat).

### Induction of colitis and MSC administration

To induce colitis, 2,4,6-trinitrobenzene-sulfonate acid (TNBS) (Sigma-Aldrich) was dissolved in 30 % ethanol to a concentration of 30 mg/kg and administered intrarectally 7 cm proximal to the anus (total volume of 300 μL) by a lubricated silicone catheter [42]. Guinea-pigs were anesthetised with isoflurane (1-4 % in O_2_) during the procedure and held at an inverted angle to prevent leakage. Sham-treated guinea-pigs underwent the same procedure without administration of TNBS. Guinea-pigs were treated with MSCs three hours after TNBS administration at the peak of tissue damage [54]. MSCs were administered by enema at a dose of 1x10^6^ cells in 300 μL of sterile PBS. Guinea-pigs were weighed and monitored daily following treatment. At 24 or 72 h after TNBS administration, animals were culled via stunning and exsanguination [55]. Segments of the distal colon were collected for histological and immunohistochemical studies.

### Tissue preparation

Colon tissues were cut along the mesenteric border, stretched and pinned flat with the mucosal side up for wholemount preparations. Tissue samples were fixed overnight at 4 °C in Zamboni’s fixative (2 % formaldehyde and 0.2 % picric acid) and subsequently washed in dimethyl sulfoxide (DMSO) (Sigma-Aldrich) (3x10min) and PBS (3x10min) to remove fixative. Samples for histology were fixed in 10 % buffered formalin solution and stored in 70 % ethanol until embedding.

### Immunohistochemistry

Immunohistochemistry was performed on wholemount preparations of the longitudinal muscle and myenteric plexus (LMMP). The preparations were dissected by removing the mucosa, submucosa and circular muscle layers to expose the myenteric plexus. LMMPs were incubated in 10 % normal donkey serum (NDS; Merck Millipore, Melbourne, Australia) at room temperature for one hour before immunolabelling. For neuronal counting, LMMPs were incubated overnight at 4 °C with primary antibodies: anti-Hu (mouse, clone 15A7.1, 1:500; Merck Millipore), anti-neuronal nitric oxide synthase (nNOS) (goat, 1:500; Novus Biologicals, Littleton, CO, USA), anti-choline acetyltransferase (ChAT) (goat, 1:500; Merck Millipore), anti-CD45 (mouse, clone IH-1, 1:200; Abcam, Melbourne, Australia) and anti-protein gene product 9.5 (PGP9.5) (rabbit, 1:500; Abcam). For analysis of immunoreactive (IR) area density, LMMP tissues were incubated with primary antibodies: anti-calcitonin gene-related peptide (CGRP) (rabbit, 1:3000; Sigma), anti-tyrosine hydroxylase (TH) (sheep, 1:1000; Merck Millipore) and anti-vesicular acetylcholine transporter (VAChT) (goat, 1:500; Merck Millipore). Tissues were washed (3x10 min PBS) and incubated for two h at room temperature with secondary antibodies: donkey anti-mouse Alexa Fluor 594 (1:200), donkey anti-goat fluorescein isothiocyanate (FITC) 488 (1:200), donkey anti-mouse FITC 488 (1:200) and donkey anti-rabbit Alexa Fluor 594 (1:200), donkey anti-sheep FITC 488 (1:200) and donkey anti-goat Alexa Fluor 647 (1:200) (all from Jackson Immunoresearch, West Grove, PA, USA). After washing, tissues were mounted on glass slides with fluorescent mounting medium (Dako North America, Inc., Carpinteria, CA, USA). For cross sections, tissues were frozen in optimal cutting temperature compound (Tissue Tek-Sakura, Tokyo, Japan) and sections were cut at a thickness of 20 μm. Cross-sections were labelled with rabbit anti-α-actin (1:1000; Abcam) followed by donkey anti-rabbit Alexa Fluor 594 (1:200) and FITC conjugated anti-human leukocyte antigen (HLA)-A,B,C (1:50; BioLegend).

### Histology

Tissues were embedded in paraffin and cut into 5 μm sections which were then deparaffinised, cleared, and rehydrated in graded ethanol. Cross sections of the colon were stained with haematoxylin and eosin and mounted on glass slides with distrene plasticizer xylene (DPX) mountant. Gross morphological damage in cross sections of the distal colon was assessed by histological grading of four parameters: mucosal flattening (0 = normal, 3 = severe flattening), occurrence of haemorrhagic sites (0 = none, 3 = frequent sites), loss of goblet cells (0 = normal, 3 = severe loss of cells), and variation of the circular muscle (0 = normal, 3 = considerable thickening of muscular layer) [32, 56].

### Imaging

Confocal microscopy was performed using an Eclipse Ti confocal laser scanning system (Nikon, Tokyo, Japan). Fluorophores were visualised using a 488 nm excitation filter for Alexa 488 or FITC and a 559 nm excitation filter for Alexa 594. Z-series images were acquired at a nominal thickness of 0.5 μm (512x512 pixels). The number of myenteric neurons Hu-IR, nNOS-IR, and ChAT-IR, as well as CD45-IR cells were counted within eight randomly captured images (total area size 2 mm^2^) per preparation at x60 magnification. CGRP-IR, TH-IR and VAChT-IR were assessed by measuring the density of immunoreactivity per area (1 mm^2^ at × 20 magnification). Image J software (National Institute of Health, Bethesda, MD, USA) was employed to convert images from RGB to greyscale 8 bit then to binary; particles were then analysed to obtain the percentage area of immunoreactivity [57]. Gross morphological damage in haematoxylin and eosin-stained colon sections was visualised using an Olympus BX53 microscope (Olympus Imaging, Melbourne, Australia) and images were captured with CellSenseTM software. Cellular imaging in vitro was performed on an Olympus IX81 inverted microscope (Olympus Imaging) using the same software.

### MSC migration assay

The distal colon was collected and high purity myenteric plexuses were isolated as described by Grundmann et al. [58]. Tissues were trypsinised for 10 min before cells were seeded into 24 well plates pre-coated with poly-L-lysine and laminin containing media. Myenteric plexuses were cultured for seven days with the media changed every second day. Medium containing lipopolysaccharide (LPS) (20 ng/mL; Sigma-Aldrich) was added to cultures for eight h to stimulate inflammatory conditions prior to the migration assay. The media conditioned by cells of the myenteric plexus were collected, pooled together and filtered through 0.2 μm pore filter to serve as a chemoattractant in the bottom well of a Boyden chamber. Controls contained unconditioned media without FBS, with equivalent FBS, or FBS with added LPS (20 ng/mL). Top wells of the Boyden chamber (pore size 8 μm, Corning Life Sciences, Tewksbury MA, USA) were loaded with 2x10^5^ BM-MSCs or AT-MSCs. After 72 h in culture, chambers were washed in PBS and cells in the bottom wells were collected by trypsinisation and counted by haemocytometry.

### Statistical analysis

Data analysis was performed using GraphPad Prism v6 (GraphPad Software, Inc., San Diego, CA, USA). Data were analysed using Student’s *t*-test (two-tailed) and one-way or two-way analysis of variance (ANOVA) for multiple group comparisons followed by Tukey’s and Sidak’s post hoc test. For all analysis *p* < 0.05 was considered significant. All data are presented as mean ± standard error of the mean (SEM).

## Results

### In vitro validation and characterisation of BM-MSCs and AT-MSCs

**Fig. 1:**
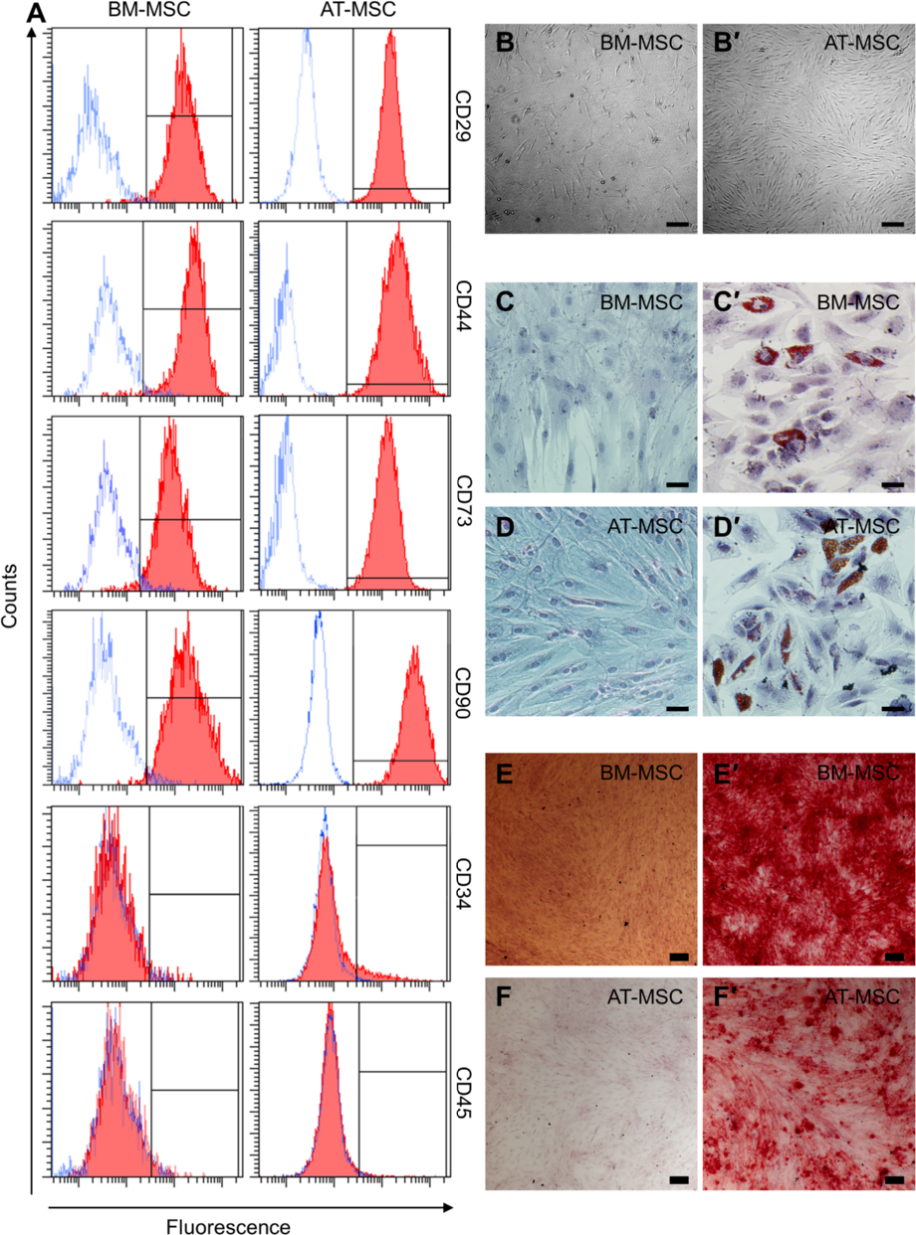
Phenotypic and functional validation of BM-MSCs and AT-MSCs. **a** BM-MSCs and AT-MSCs analysed for cell surface antigen expression of known positive (CD29, CD44, CD73, and CD90) and negative (CD34 and CD45) MSC markers. Red closed histograms represent MSCs labelled with antibodies against the surface antigen indicated on the *right hand side* of each row. Blue open histograms show isotype controls. BM-MSCs **b** and AT-MSCs *b*′ adhered to plastic with a perceptible appearance typical of MSCs in culture. Scale bar = 200 μm. BM-MSCs and AT-MSCs cultured without **c**-**d** and with *c*′-*d*′ adipogenesis differentiation medium for 14 days and stained with Oil red O. Scale bar = 50 μm. BM-MSCs and AT-MSCs cultured without **e**-**f** and with *e*′-*f*′ osteogenesis differentiation medium for 21 days and stained with Alizarin red S. Scale bar = 200 μm. *BM-MSCs* bone marrow-derived mesenchymal stem cells, *AT-BMCs* adipose tissue-derived mesenchymal stem cells

All experiments were conducted using MSCs cultured until the fourth passage. Flow cytometry was used to validate the immunophenotype of MSCs. Cell surface expression of positive MSC markers CD29, CD44, CD73 and CD90 was observed in BM-MSCs (98.5 %, 98.8 %, 98.0 %, and 98.9 %, respectively) and AT-MSCs (99.9 %, 99.8 %, 99.9 %, and 99.6 % respectively) (Fig. 1a). BM-MSCs demonstrated negligible expression of non-MSC markers CD34 (2.5 %) and CD45 (3.6 %). In AT-MSCs, the expression of CD34 was low (10.3 %) while CD45 was negligible (1.6 %). Thus, the immunophenotype of BM-MSCs and AT-MSCs was in compliance with MSC definition [2].

MSCs adhered to plastic and proliferated to form monolayer cultures (Fig. 1b-*b*′). In addition, BM-MSCs appeared more sparsely distributed than AT-MSCs. The multipotent potential of MSCs was assessed by exposing cells to adipogenic and osteogenic differentiation media. MSCs stained positive with Oil Red O indicative of successful induction to adipocytes with lipid filled vacuoles (Fig. 1c-*d*′). Confirmation of differentiation to osteogenic lineage was revealed by Alizarin red S staining of calcium deposition (Fig. 1e-*f*′). Both BM-MSCs and AT-MSCs exhibited multipotency and, therefore, were considered bona fide MSCs.

**Fig. 2:**
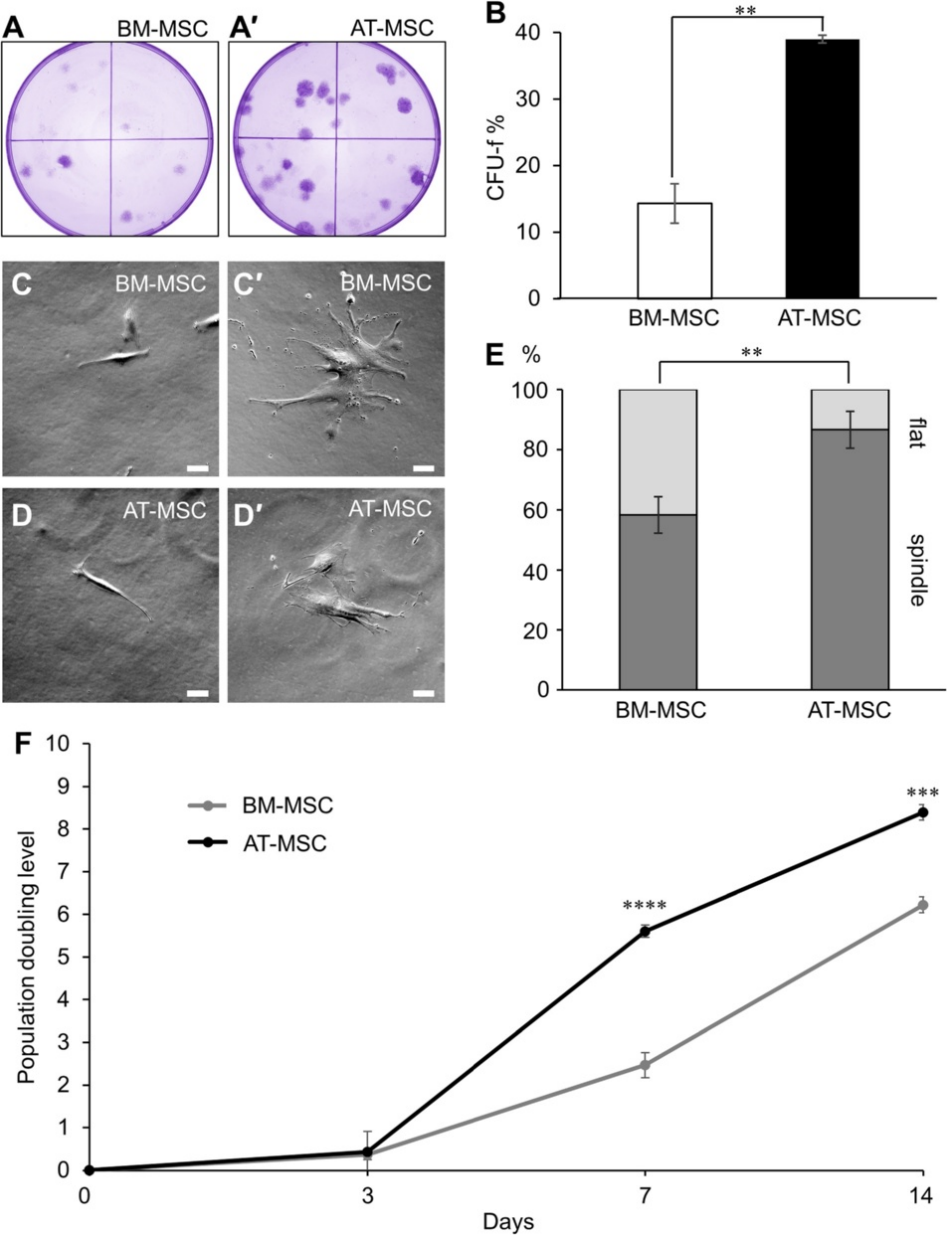
In vitro clonogenicity, morphology, and growth kinetics of MSCs. Clonogenicity of BM-MSCs **a** and AT-MSCs *a′* determined by a colony forming unit-fibroblast (CFU-f) assay (n = 3 independent cultures/group). **b** CFU-f counts quantified as a percentage of the total viable cells seeded. **c**-*d*′) Morphological subpopulations exhibited by BM-MSCs **c**-*c*′) and AT-MSCs **d**-*d*′ in culture. MSC morphology defined according to the presence of long thin spindles (‘spindle’: **c**-**d** or flat cells with atypical processes (‘flat’: *c*′-*d*′) (scale bar = 50 μm). **e** Quantitative analysis of MSC morphological types. Data expressed as a percentage of the total cell number in each population (n = 6 independent cultures/group). **f**) The population doubling level (PDL) of proliferating MSCs recorded at 3, 7 and 14 days after seeding (n = 3 independent cultures/group/time point). **p < 0.01, ***p < 0.001, ****p < 0.0001. MSCs mesenchymal stem cells, *BM-MSCs* bone marrow-derived mesenchymal stem cells, *AT-MSCs* adipose tissue-derived mesenchymal stem cells

A CFU-f assay was performed to compare the clonogenicity between BM-MSCs and AT-MSCs. The percentage of MSCs capable of developing into colonies was greater in AT-MSC cultures (39.0 ± 0.6 %) compared to those of BM-MSCs (14.3 ± 3.0 %, *p* < 0.01) after two weeks (Fig. 2a-*a*′, b, n = 3 independent cultures/group). Subpopulations of MSCs were quantified by the morphological properties of their cell bodies in vitro (Fig. 2c-*d*′). Two prominent morphological types were exhibited in MSC cultures consisting of cells with long thin ‘spindle’ shapes (Fig. 2c, d) and ‘flat’ cells with irregular processes (Fig. 2*c*′, *d*′). Cell populations with a ‘spindle’ morphology were more readily exhibited by AT-MSCs (86.7 ± 6.1 %) than BM-MSCs (58.3 ± 6.0 %, *p* < 0.01, n = 6 independent cultures/group, Fig. 2e). In the contrary, a higher population of cells with a ‘flat’ morphology was observed in BM-MSCs (41.7 ± 6.0 %) in comparison to AT-MSCs (13.3 ± 6.1 %, *p* < 0.01). The growth kinetics of MSCs were quantified over 14 days in culture and an assessment was made on the population doubling rate (Fig. 2f). No difference in the PDL was observed between BM-MSCs (0.4 ± 0.1) and AT-MSCs (0.4 ± 0.5) after three days (n = 3 independent cultures/group/time point). Higher PDL was observed in AT-MSC compared to BM-MSC cultures at day 7 (5.6 ± 0.1 vs 2.5 ± 0.3, *p* < 0.0001) continuing to day 14 (8.4 ± 0.2 vs 6.2 ± 0.2, *p* < 0.001). Thus, the in vitro AT-MSC phenotype exhibited characteristics associated with superior cellular expansion.

### BM-MSCs and AT-MSCs comparably ameliorate histological damage and weight loss associated with TNBS-induced colitis

**Fig. 3:**
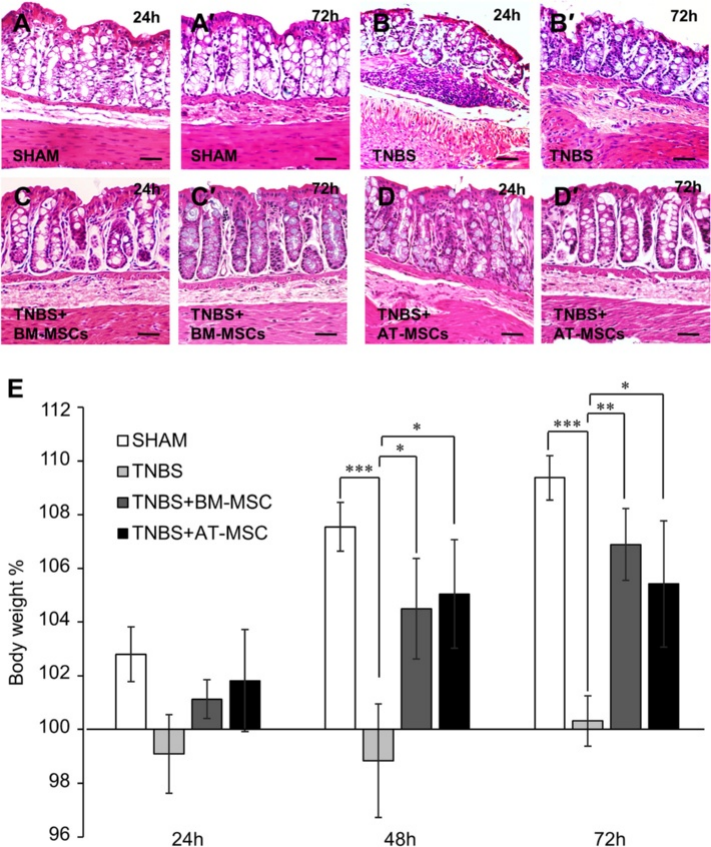
Effects of MSC treatment on histological changes and body weight in colitis. Colonic structure assessed via haematoxylin and eosin staining of cross sections from tissues collected at 24 h **a**-**d** and 72 h *a*′-*d*′ post TNBS administration. Scale bar = 50 μm. **e** Body weight recorded at 24, 48 and 72 h after TNBS administration and expressed as the change from baseline measurements. *p < 0.05, **p < 0.01, ***p < 0.001, n = 4 animals/group/time point. *MSC* mesenchymal stem cell, TNBS 2,4,6-trinitrobenzene-sulfonate acid

Gross morphological damage was assessed in haematoxylin and eosin-stained cross sections of the colon. No damage was observed in sham-treated guinea-pigs (histological score = 0, Fig. 3a-*a*′). At 24 and 72 h following the induction of TNBS-induced colitis, changes to the colonic architecture were observed including flattening of the mucosa, haemorrhagic sites, loss of goblet cells and altered presentation of the circular muscle layer (histological score = 2–3) (Fig. 3b-*b*′). These changes were attenuated in both BM-MSC- and AT-MSC-treated animals at 24 h and 72 h (histological score = 0–1, Fig. 3c-*c*′, d-*d*′).

The body weight of guinea-pigs was recorded before and after treatment at 24, 48 and 72 h (Fig. 3e, Table 1, n = 4 animals/group/time point). No differences in weight were observed between treatment groups after 24 h. By 48 and 72 h, the body weight of the TNBS group was lower compared to the sham group (both *p* < 0.001). The reduction in body weight was ameliorated by both BM-MSC (48 h, *p* < 0.05 and 72 h, *p* < 0.01) and AT-MSC treatments (48 h and 72 h *p* < 0.05). Therefore, BM-MSCs and AT-MSCs were equally effective in attenuating colitis-induced weight loss.

**Table 1:** Effects of mesenchymal stem cells derived from bone marrow and adipose tissue on body weight (%) in TNBS-induced colitis

	Sham	TNBS	TNBS + BM-MSC	TNBS + AT-MSC
24 h	102.8 ± 1.0	99.1 ± 1.5	101.1 ± 0.7	101.8 ± 1.9
48 h	107.5 ± 0.9	98.8 ± 2.1 †††	104.5 ± 1.9 *	105.0 ± 2.0 *
72 h	109.4 ± 0.8	100.3 ± 0.9 †††	106.9 ± 1.3 **	105.4 ± 2.4 *

*TNBS*, 2,4,6-trinitrobenzene sulfonic acid, *BM-MSC* bone marrow-derived MSC, *AT-MSC* adipose tissue derived MSC

*p < 0.05, **p < 0.01, significantly different to TNBS; †††*p* < 0.001, significantly different to sham

### BM-MSCs are more efficient than AT-MSCs in attenuating leukocyte infiltration to the level of the myenteric ganglia

**Fig. 4:**
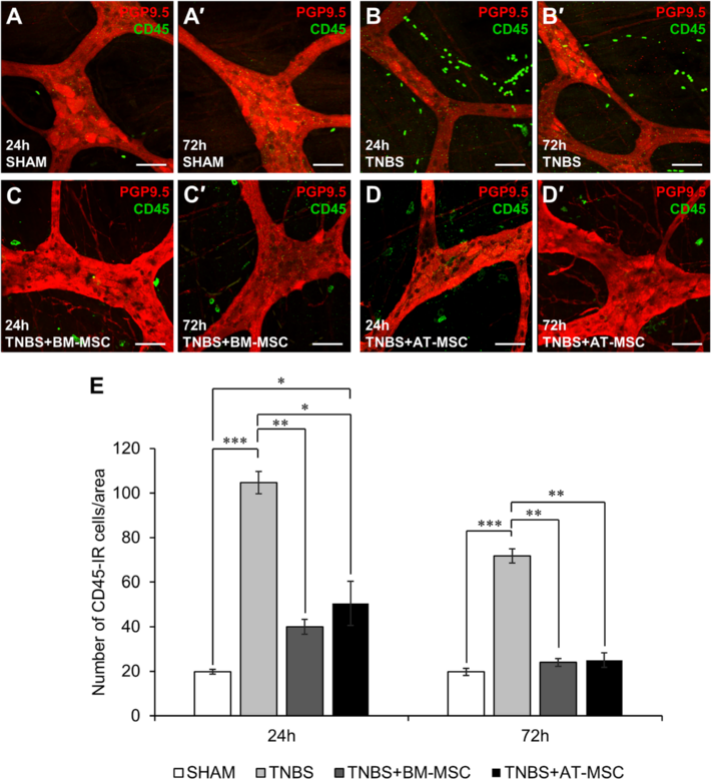
Effects of MSCs on leukocyte infiltration to the myenteric plexus. **a**-*d*′ CD45-IR leukocytes (green) visualised on the level of myenteric neurons labelled with anti-PGP9.5 (red) by confocal microscopy in LMMP wholemounts prepared from colon collected at 24 **a**-**d** and 72 h *a*′-*d*′ post treatment. Scale bar = 50 μm. **e** CD45-IR leukocytes quantified in a 2 mm^2^ area of the myenteric plexus in the colon. *p < 0.05, **p < 0.01, ***p < 0.001, n = 4 animals/group/time point. *MSCs* mesenchymal stem cells, *LMMP* longitudinal muscle and myenteric plexus

The number of leukocytes in proximity to the myenteric plexus were quantified in LMMP preparations of the guinea-pig colon (Fig. 4a-*d*′, n = 4 animals/group/time point). Elevated leukocyte counts (cells/area) were observed in TNBS groups at 24 h (104.8 ± 5.0) and 72 h (71.8 ± 3.2) compared to shams (24 h: 19.8 ± 1.0 and 72 h: 19.8 ± 1.6, both *p <* 0.001; Fig. 4e). At 24 h, elevated leukocyte levels were attenuated by BM-MSC (40.0 ± 3.3, *p <* 0.01) and AT-MSC (50.5 ± 9.9, *p <* 0.05) treatments. However, the number of leukocytes was still elevated in AT-MSC-treated animals in comparison to sham (*p <* 0.05). By 72 h, TNBS-induced leukocyte infiltration was mitigated by BM-MSC (24.0 ± 1.7, *p <* 0.01) and AT-MSC (25.0 ± 3.2, *p <* 0.01) treatments to levels comparable to shams. These results demonstrate that both MSC types can attenuate plexitis; however BM-MSCs appear to act faster.

### BM-MSCs and AT-MSCs have comparable efficacy for attenuating inflammation-induced enteric neuropathy

**Fig. 5:**
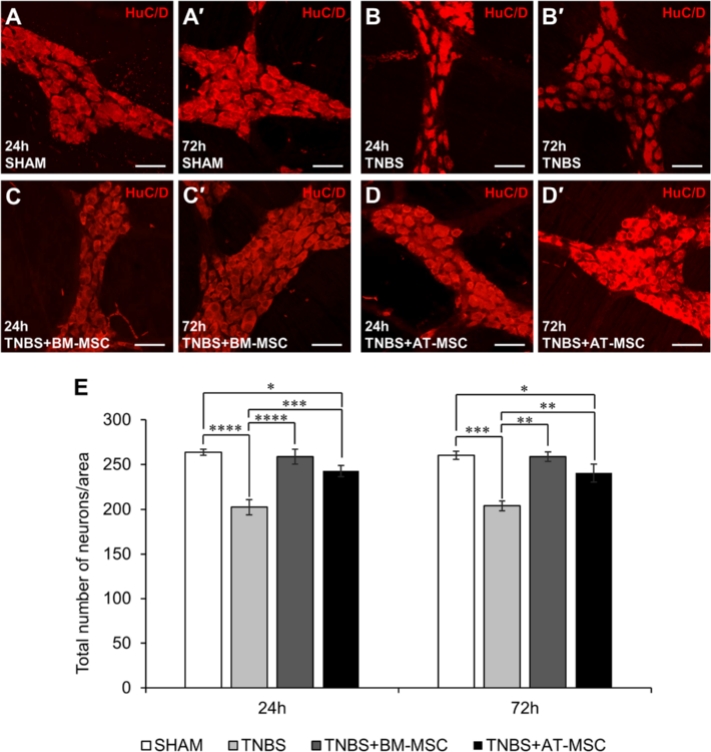
Effects of BM-MSCs and AT-MSCs on the total number of myenteric neurons. **a**-*d*′ Neuronal cell bodies in the myenteric plexus were labelled with the pan neuronal marker anti-HuC/D antibody at 24 **a**-**d** and 72 h *a*′-*d*′ post treatment. Scale bar = 50 μm. **e** The total number of neuronal bodies were quantified within a 2 mm^2^ area of the myenteric plexus. *p < 0.05, **p < 0.01, ***p < 0.001, ****p < 0.0001, n = 4 animals/group/time point. *BM-MSCs* bone marrow-derived mesenchymal stem cells, *AT-MSCs* adipose tissue-derived mesenchymal stem cells

The pan neuronal marker HuC/D was used to assess the neuroprotective efficacy of MSCs in wholemount LMMP preparations (Fig. 5a-*d*′, n = 4 animals/group/time point). In comparison to sham, the administration of TNBS resulted in neuronal loss at 24 h (*p* < 0.0001) which persisted at 72 h (*p* < 0.001, Fig. 5e, Table 2). The loss of neurons was ameliorated at both time points by BM-MSC (24 h: *p* < 0.0001 and 72 h: *p* < 0.01) and AT-MSC treatments (24 h: *p* < 0.001 and 72 h: *p* < 0.01). However, AT-MSC treatment was less effective and differences to the sham groups were observed at 24 and 72 h (both *p* < 0.05, Fig. 5e).

**Table 2:** Effects of mesenchymal stem cells derived from bone marrow and adipose tissue on myenteric neurons in TNBS-induced colitis

	Sham	TNBS	TNBS + BM-MSC	TNBS + AT-MSC
	Total number of myenteric neurons/2 mm^2^			
24 h	263.8 ± 3.5	202.5 ± 8.4 ††††	258.8 ± 2.9 ****	242.8 ± 6.2 *** †
72 h	262.0 ± 5.1	204.0 ± 5.4 †††	258.8 ± 7.6 **	240.5 ± 10.0 ** †
	Total number of nNOS-IR neurons/2 mm^2^			
24 h	52.3 ± 1.8	70.0 ± 4.9 †††	55.0 ± 3.2 **	53.5 ± 2.6 **
72 h	52.5 ± 1.0	70.5 ± 3.2 †††	55.3 ± 2.7 **	57.3 ± 1.7 *
	Proportion of nNOS-IR neurons/2 mm^2^ (%)			
24 h	19.9 ± 0.9	34.8 ± 2.8 ††††	21.3 ± 1.2 ****	22.1 ± 1.1 ****
72 h	20.2 ± 0.3	34.6 ± 1.8 ††††	21.4 ± 1.3 ****	23.9 ± 0.7 ***
	Total number of ChAT-IR neurons/2 mm^2^			
24 h	163.5 ± 2.1	111.3 ± 5.7 ††††	148.3 ± 4.6 ****	144.5 ± 4.9 *** †
72 h	157.8 ± 6.3	112.3 ± 1.7 ††††	147.5 ± 7.3 ****	145.3 ± 3.4 ***
	Proportion of ChAT-IR neurons/2 mm^2^ (%)			
24 h	62.0 ± 1.4	55.1 ± 2.9	57.3 ± 1.6	59.5 ± 0.9
72 h	59.1 ± 0.9	55.2 ± 2.3	57.1 ± 3.0	60.7 ± 2.5

*TNBS* 2,4,6-trinitrobenzene sulfonic acid, *BM-MSC* bone marrow-derived MSC, *AT-MSC* adipose tissue-derived MSC, *MSC* mesenchymal stem cell, *nNOS* neuronal nitric oxide synthase, *ChAT* choline acetyltransferase, *IR* immunoreactive

*p < 0.05, **p < 0.01, ***p < 0.001, ****p < 0.0001, significantly different to TNBS; †*p* < 0.05, †††*p* < 0.001, ††††*p* < 0.0001, significantly different to sham

**Fig. 6:**
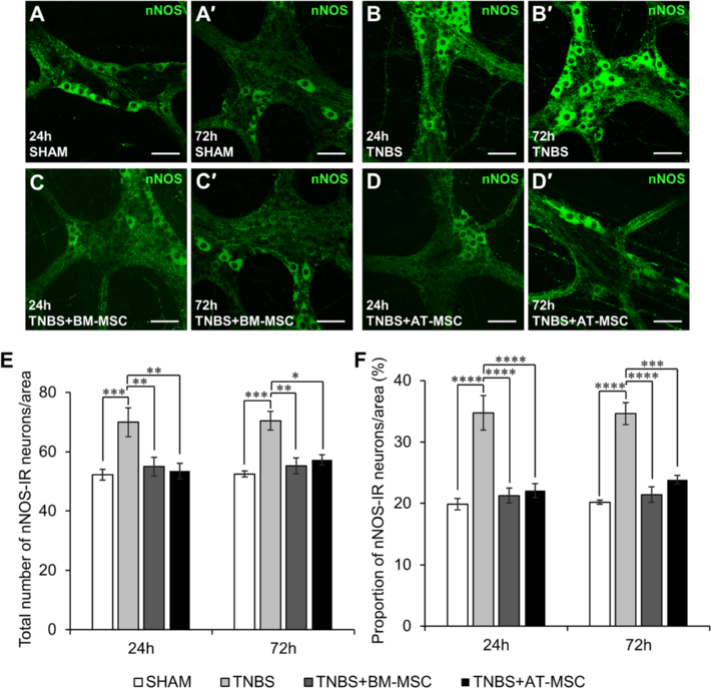
Effects of BM-MSCs and AT-MSCs on nitrergic myenteric neurons. **a**-*d*′ Nitrergic (nNOS-IR) neurons were visualised in the myenteric plexus at 24 **a**-**d** and 72 h *a*′-*d*′. Scale bar = 50 μm. The total number of nNOS-IR neurons **e** and the proportion of nNOS-IR neurons to the total number of HuC/D-IR neurons **f** were quantified within a 2 mm^2^ area of the myenteric plexus in the guinea-pig colon. *p < 0.05, **p < 0.01, ***p < 0.001, ****p < 0.0001, n = 4 animals/group/time point. *BM-MSCs* bone marrow-derived mesenchymal stem cells, *AT-MSCs* adipose tissue-derived mesenchymal stem cells

Inhibitory and excitatory neurons, defined by nNOS-IR and ChAT-IR respectively, were quantified within the myenteric ganglia (Figs. 6a-*d*′ and 7a-*d*′, Table 2, n = 4 animals/group/time point). Increased numbers (both *p* < 0.001) and proportions (both *p* < 0.0001) of nNOS-IR neurons were observed in myenteric ganglia from TNBS groups at 24 and 72 h compared to sham (Fig. 6e-f, Table 2). BM-MSC and AT-MSC treatments attenuated these changes in the total number (BM-MSC: 24 h and 72 h, *p* < 0.01 and AT-MSC: 24 h, *p* < 0.01 and 72 h, *p* < 0.05) and proportion of nNOS-IR neurons at both time points (BM-MSC: 24 h and 72 h, *p* < 0.0001; AT-MSCs: 24 h, *p* < 0.0001 and 72 h, *p* < 0.001). The total numbers of ChAT-IR neurons were decreased at both 24 and 72 h after TNBS administration compared to sham (*p <* 0.0001 for both time points) (Fig. 6e, Table 2). The loss of ChAT-IR neurons was attenuated at 24 and 72 h by treatments with BM-MSCs (*p <* 0.0001) and AT-MSCs (*p <* 0.001 for both time points). However, the number of ChAT-IR neurons were still less than sham after AT-MSC treatment at 24 h (*p <* 0.05). Quantification of the proportion of ChAT-IR neurons revealed no differences between groups (Fig. 6f, Table 2). Thus, although both BM-MSCs and AT-MSCs were effective in attenuating neuronal loss and changes in nNOS and ChAT immunoreactivity, AT-MSCs were less efficacious compared to BM-MSCs in treating neuropathy.

**Fig. 7:**
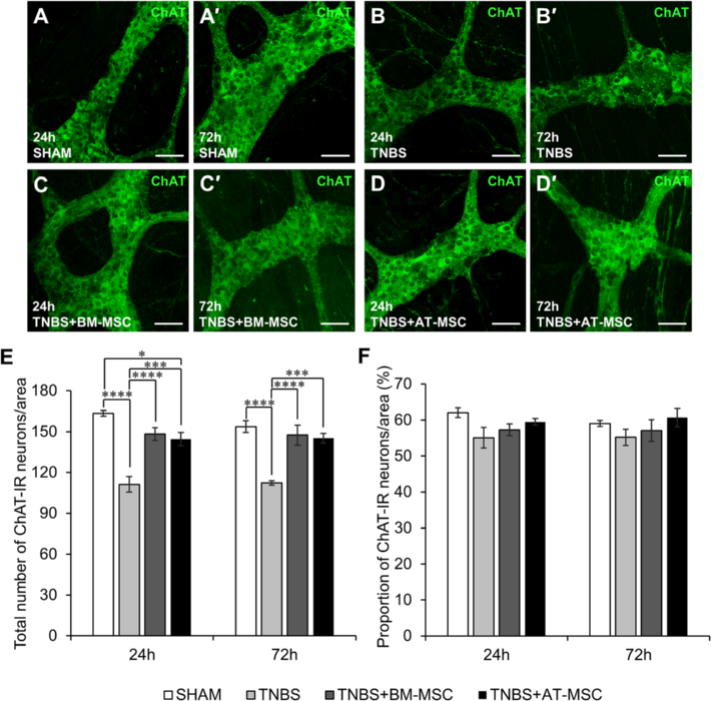
Effects of BM-MSCs and AT-MSCs on cholinergic myenteric neurons. **a**-*d*′ Cholinergic (ChAT-IR) neurons in the myenteric plexus at 24 **a**-**d** and 72 h *a*′-*d*′. Scale bar = 50 μm. The total number of ChAT-IR neurons **e** and the proportion of ChAT-IR neurons to the total number of HuC/D-IR neurons **f** were quantified within a 2 mm^2^ area of the myenteric plexus in the guinea-pig colon. *p < 0.05, ***p < 0.001, ****p < 0.0001, n = 4 animals/group/time point. *BM-MSCs* bone marrow-derived mesenchymal stem cells, *AT-MSCs* adipose tissue-derived mesenchymal stem cells, *ChAT* choline acetyltransferase, *IR* immunoreactive

### BM-MSCs and AT-MSCs mitigate the loss of nerve fibres in the inflamed colon

**Fig. 8:**
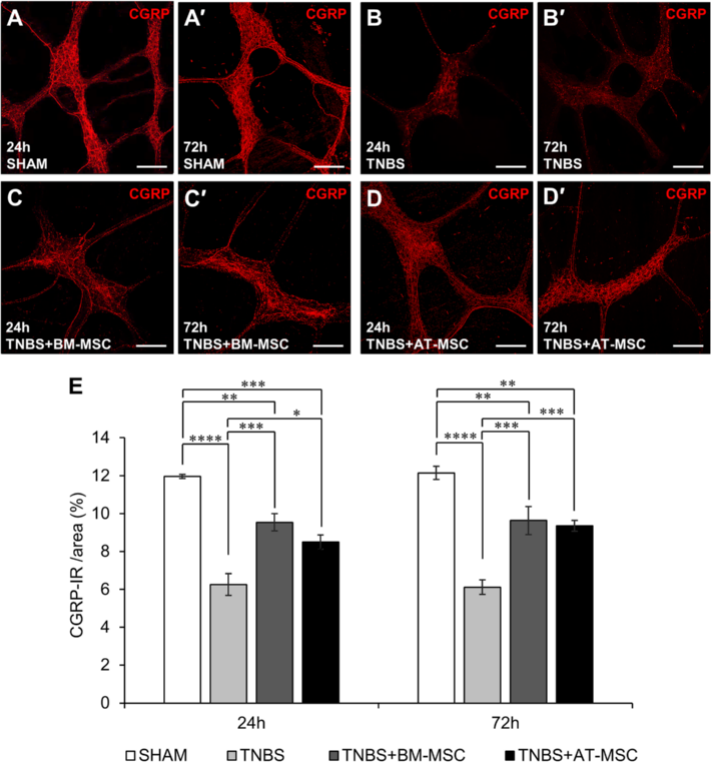
Effects of BM-MSCs and AT-MSCs on CGRP-IR nerve fibres in the myenteric plexus. **a**-*d*′ CGRP-IR in the myenteric plexus at 24 h **a**-**d** and 72 h *a*′-*d*′. Scale bar = 100 μm. **e** Area percentage quantification of CGRP-IR within a 1 mm^2^ area of the myenteric plexus in the guinea-pig colon. *p < 0.05, **p < 0.01, ***p < 0.001, ****p < 0.0001, n = 3 animals/group/time point. *BM-MSCs* bone marrow-derived mesenchymal stem cells, *AT-MSCs* adipose tissue-derived mesenchymal stem cells, *CGRP* calcitonin gene-related peptide, *IR* immunoreactive

**Fig. 9:**
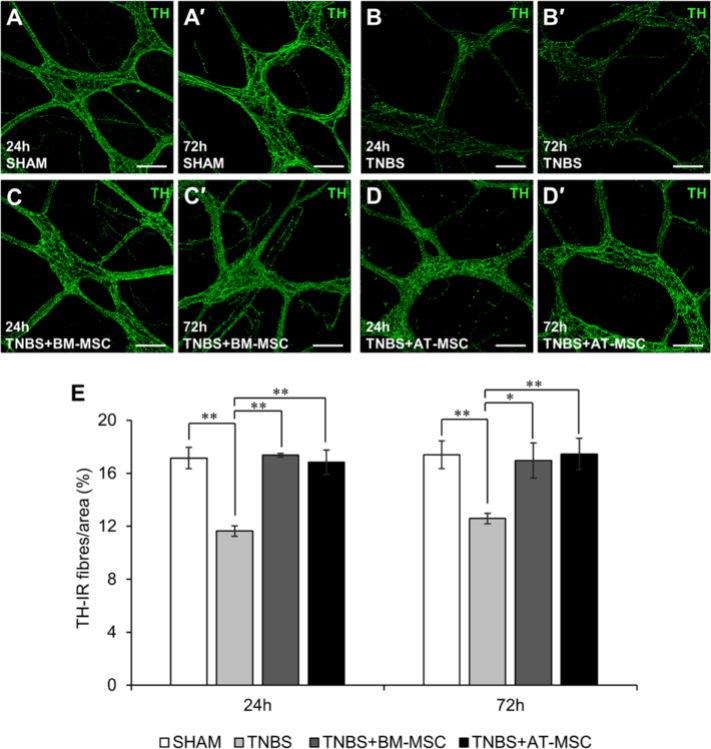
Effects of BM-MSCs and AT-MSCs on TH-IR nerve fibres in the myenteric plexus. **a**-*d*′ TH-IR nerve fibres in the myenteric plexus at 24 h **a**-**d** and 72 h *a*′-*d*′. Scale bar = 100 μm. **e** Area percentage quantification of TH-IR nerve fibres within a 1 mm^2^ area of the myenteric plexus in the guinea-pig colon. *p < 0.05, **p < 0.01, n = 3 animals/group/time point. *BM-MSCs* bone marrow-derived mesenchymal stem cells, *AT-MSCs* adipose tissue-derived mesenchymal stem cells, *TH* tyrosine hydroxylase, *IR* immunoreactive

**Fig. 10:**
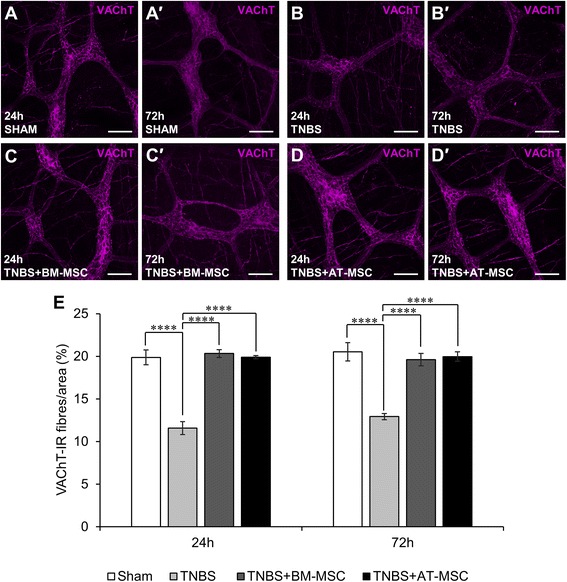
Effects of BM-MSCs and AT-MSCs on VAChT-IR nerve fibres in the myenteric plexus. **a**-*d*′ VAChT-IR nerve fibres in the myenteric plexus at 24 h **a**-**d** and 72 h *a*′-*d*′. Scale bar = 100 μm. **e** Area percentage quantification of VAChT-IR nerve fibres within a 1 mm^2^ area of the myenteric plexus in the guinea-pig colon. ****p < 0.0001, n = 3 animals/group/time point. *BM-MSCs* bone marrow-derived mesenchymal stem cells, *AT-MSCs* adipose tissue-derived mesenchymal stem cells, *VAChT* vesicular acetylcholine transporter, *IR* immunoreactive

Immunoreactivity for CGRP (sensory), TH (sympathetic) and VAChT (cholinergic) was assessed within the myenteric plexus in LMMP preparations (Figs. 8a-*d*′, 9a-*d*′ and 10a-*d*′, Table 3). At 24 and 72 h, TNBS administered groups exhibited decreased density of CGRP immunoreactivity (Fig. 8e, *p* < 0.0001 for both time points) compared to shams. At these time points, BM-MSC and AT-MSC treatments attenuated the loss of CGRP immunoreactivity (BM-MSC: *p* < 0.001 for both time points; AT-MSC: 24 h, *p* < 0.05 and 72 h *p* < 0.001). However, the loss of CGRP-IR was not prevented to the levels of sham groups by BM-MSCs (*p* < 0.01 for both time points) and AT-MSCs (24 h, *p* < 0.001 and 72 h, *p* < 0.01) treatments. Similar loss in density was observed after TNBS administration in TH-IR (Fig. 9e, both *p* < 0.01) and VAChT-IR nerve fibres (Fig. 10e, both *p* < 0.0001) compared to shams. BM-MSC and AT-MSC treatments ameliorated the loss of TH-IR (BM-MSC: 24 h, *p* < 0.01 and 72 h, *p* < 0.05; AT-MSC: *p* < 0.01 for both time points) and VAChT-IR (all *p* < 0.0001) nerve fibres. BM-MSCs and AT-MSCs were equally efficacious in attenuating the loss of nerve fibre immunoreactivity.

**Table 3:** Effects of mesenchymal stem cells derived from bone marrow and adipose tissue on nerve fibre density in TNBS-induced colitis

	Sham	TNBS	TNBS + BM-MSC	TNBS + AT-MSC
	Level of CGRP immunoreactivity/1 mm^2^ (%)			
24 h	12.0 ± 0.1	6.3 ± 0.6 ††††	9.5 ± 0.5 *** ††	8.5 ± 0.4 * †††
72 h	12.2 ± 0.4	6.1 ± 0.4 ††††	9.6 ± 0.7 *** ††	9.4 ± 0.3 *** ††
	Density of TH-IR nerve fibres/1 mm^2^ (%)			
24 h	17.2 ± 0.8	11.6 ± 0.4 ††	17.4 ± 0.1 **	16.8 ± 0.9 **
72 h	17.4 ± 1.1	12.6 ± 0.4 ††	17.0 ± 1.3 *	17.5 ± 1.2 **
	Density of VAChT-IR nerve fibres/1 mm^2^ (%)			
24 h	19.9 ± 0.9	11.6 ± 0.8 ††††	20.3 ± 0.5 ****	19.9 ± 0.2 ****
72 h	20.5 ± 1.1	12.9 ± 0.4 ††††	19.6 ± 0.7 ****	20.0 ± 0.6 ****

*TNBS* 2,4,6-trinitrobenzene sulfonic acid, *BM-MSC* bone marrow derived mesenchymal stem cells, *AT-MSC* adipose tissue derived mesenchymal stem cells, *CGRP* calcitonin gene-related peptide, *TH* tyrosine hydroxylase, *VAChT* vesicular acetylcholine transporter, *IR* immunoreactive

*p < 0.05, **p < 0.01, ***p < 0.001, ****p < 0.0001, significantly different to TNBS; ††*p* < 0.01, †††*p* < 0.001, ††††*p* < 0.0001, significantly different to sham

### BM-MSCs and AT-MSCs migrate to the myenteric plexus

**Fig. 11:**
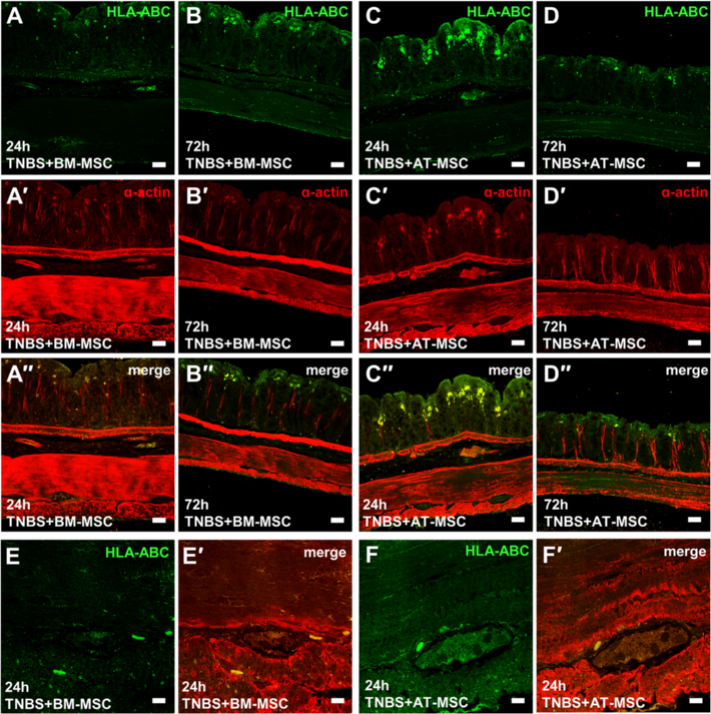
In vivo migration of BM-MSCs and AT-MSCs. **a**-*d*′′ Cross-sections of the guinea-pig colon after treatment with BM-MSCs **a**-**b** and AT-MSCs **c**-**d** labelled with anti-HLA **a**-**d** to detect human MSCs and anti-α-actin to visualise smooth muscle *a*′-*d*′. Scale bar = 50 μm. *e*′-*f*′ High magnification confocal images (x100) of myenteric ganglia from BM-MSC **e**-*e*′ and AT-MSC**-**treated guinea-pigs **f**-*f*′. Scale bar = 10 μm. *BM-MSCs* bone marrow-derived mesenchymal stem cells, *AT-MSCs* adipose tissue-derived mesenchymal stem cells, *HLA* human leukocyte antigen

BM-MSCs and AT-MSCs were detected in cross-sections of the colon at 24 h and 72 h as defined by HLA-IR (Fig. 11a-*d*′′). Predominantly, MSCs were observed within the mucosa and to a lesser extent in the submucosa. A relatively low number of HLA-IR cells was observed in the muscle layers. High magnification confocal images (x100) confirmed the presence of HLA-IR cells at the level of the myenteric ganglia in both BM-MSC (Fig. 11e-*e*′) and AT-MSC (Fig. 11f-*f*′) treated groups.

**Fig. 12:**
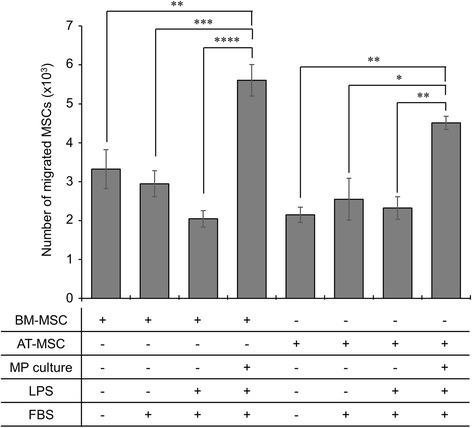
In vitro migration of BM-MSCs and AT-MSCs. Quantification of MSC migration towards the conditioned media of cultured myenteric plexus (MP) cells pre-stimulated with LPS in a modified Boyden chamber assay. **p < 0.01, ***p < 0.001, ****p < 0.0001, n = 4 independent cultures/group. *BM-MSCs* bone marrow-derived mesenchymal stem cells, *AT-MSCs* adipose tissue-derived mesenchymal stem cells, *LPS* lipopolysaccharide

To assess the migration of MSCs to the inflamed myenteric plexus, an in vitro migration assay was performed using LPS stimulated neurons as a chemoattractant (Fig. 12). BM-MSCs and AT-MSCs had a higher affinity to migrate to the conditioned medium of LPS-stimulated myenteric neurons (BM-MSC: 5.6 ± 0.4 × 10^3^; AT-MSC: 4.5 ± 0.2 × 10^3^) compared to control media without FBS (BM-MSC: 3.3 ± 0.5 × 10^3^; AT-MSC: 2.2 ± 0.2 × 10^3^, *p* < 0.01 for both) or supplemented with FBS (BM-MSC: 2.9 ± 0.3 × 10^3^, *p* < 0.001; AT-MSC: 2.6 ± 0.5 × 10^3^, *p* < 0.05) and medium containing the same amount of LPS (20 ng/mL) as the conditioned medium of LPS-stimulated myenteric neurons (BM-MSC: 2.0 ± 0.2 × 10^3^, *p* < 0.0001; AT-MSC: 2.3 ± 0.3 × 10^3^, *p* < 0.01). No differences in migration were observed between BM-MSCs and AT-MSCs.

## Discussion

This is the first study comparing the neuroprotective efficacy of AT-MSCs and BM-MSCs in a model of colitis. In vitro, AT-MSCs possessed a superior phenotype for cellular expansion. Both AT-MSCs and BM-MSCs demonstrated therapeutic efficacy in the amelioration of weight loss, histopathology, leukocyte infiltration to the myenteric plexus, neuronal loss, altered neurochemical expression, and damage to nerve fibres. However, AT-MSCs appeared less effective in the attenuation of plexitis, neuropathy, and reduction in ChAT immunoreactivity.

MSCs used in this study were validated according to the guidelines of the International Society for Cellular Therapy [2]. BM-MSCs and AT-MSCs displayed a typical surface marker phenotype including positive expression of CD29, CD44, CD73, and CD90 in addition to negligible expression of CD45. AT-MSCs exhibited low positive expression of CD34; however, it is now accepted that CD34^+^ MSCs are a common subpopulation residing in the adipose tissue [59]. MSCs from both sources demonstrated multipotency by differentiation into adipocytes and osteocytes when cultured in media supplemented with appropriate differentiation factors. Both cell types were plastic adherent and proliferated to form colonies, a definitive characteristic of MSCs.

In vitro expansion of MSC cultures is fundamental to obtain appropriate numbers for therapeutic application. Our results show that after an initial ~3-day lag period, AT-MSCs proliferate at a higher rate than BM-MSCs yielding greater quantities of cells. Other studies have shown similar differences in proliferation exhibited by BM-MSCs and AT-MSCs [60–62]. The ability of MSCs to form colonies is reflective of their expansive capacity [63]. The CFU-f assay is regarded as the gold standard for identifying clonogenic MSCs [64]. Both MSC types were capable of developing colony forming units. However, AT-MSCs produced more than twice the number of colony forming units compared to BM-MSCs. Similar results have been reported in multiple human MSC lines [63].

The heterogeneous in vitro characteristics of BM-MSCs and AT-MSCs were further elucidated by defining morphological subpopulations. AT-MSC cultures were dominated by cells with ‘spindle’ morphology. Inversely, the proportion of ‘flat’ MSCs was greater in BM-MSC cultures. In agreement with these results, rat AT-MSCs have also been observed to contain higher populations of ‘spindle’ shaped cells with higher proliferative capacity [65]. Evidence of a direct relationship between MSC morphology and expansive characteristics arise from the parallels of decreasing proliferation and clonogenicity [66, 67] and increasing populations of ‘flat’ MSCs [68] over subsequent passaging. Nonetheless, differences in proliferation and clonogenicity between BM-MSCs and AT-MSCs are observed consistently at equivalent passages [60–63]. In our study, colony forming units were predominantly populated by ‘spindle’ MSCs suggesting a link between morphology and clonogenicity. It should be noted that ‘flat’ MSCs were also observed to be clonogenic; however, these colonies were rarely detected by the CFU-f assay due to their inferior proliferative nature. Nonetheless, these data collectively suggest that morphological subpopulations may be indicative of expansion potential, which could be useful information when propagating MSC therapies in the clinic. Due to the great number of MSCs required for human therapy, the in vitro phenotype is a crucial consideration to determine the favourable source of MSC treatment in the clinic. AT-MSCs were shown to exhibit superior in vitro properties. However, the in vivo therapeutic efficacy of MSCs from different sources also requires attention.

In our study, the efficacy of BM-MSCs and AT-MSCs was assessed in an in vivo model of intestinal inflammation induced by administering TNBS which initiated an immune response to hapten modified autologous proteins [69]. Lack of weight gain is commonly observed in this model and is reflective of the inflammatory state [70, 71]. This effect was comparably attenuated in guinea-pigs treated with BM-MSCs and AT-MSCs. The histopathological severity of experimental colitis has been evaluated to determine the effectiveness of MSC treatments [26, 30, 72, 73]. In our study, both MSC treatments similarly prevented disruption to the epithelial lining, inflammatory infiltrate and changes to the colonic architecture. Previous comparisons of allogeneic rat MSCs from these tissue sources in experimental colitis are in agreement with these observations [31]. While MSCs from both sources were seemingly equally beneficial in attenuating the manifestations of TNBS-induced colitis, we further investigated their therapeutic efficacy for the treatment of enteric neuropathy associated with intestinal inflammation.

The increased number of leukocytes in proximity to myenteric ganglia upon administration of TNBS, indicative of plexitis, was prevented by both MSC treatments. However, leukocyte numbers were still elevated after AT-MSC treatment compared to shams at early stages of inflammation which suggests that AT-MSCs exert their immunomodulatory effects more slowly than BM-MSCs. Previous studies in various pathologies appear to be in agreement that BM-MSCs are superior to AT-MSCs in preventing leukocyte infiltration and inflammation [21–24]. The infiltration of leukocytes to the myenteric plexus in the resected bowel of Crohn’s disease patients is predictive of inflammatory relapse requiring repeated surgery [45, 46]. Enteric neurons express receptors for inflammatory mediators, activation of which causes substantial excitation in enteric neurons [74]. Inflammation-induced neuronal death and axonal damage leads to changes in neurally-controlled intestinal functions [75, 76].

Neuronal loss was observed in animals administered with TNBS in our study consistent with previous reports [40–42]. Both BM-MSCs and AT-MSCs attenuated neuronal loss in animals with colitis, however BM-MSCs were more effective compared to AT-MSCs. In the myenteric plexus, the two major subpopulations of excitatory and inhibitory muscle motor and interneurons, ChAT-IR (cholinergic) and nNOS-IR (nitrergic), were further investigated [77]. Changes in the neurochemical coding of these neurons are associated with altered coordination of muscular contractions and dysmotility in humans and animals [78–80]. Intestinal dysmotility is a symptom of IBD; however, it may also contribute to disease progression through dysfunctional propulsive clearance of enterotoxins that promote additional inflammatory bouts [81]. Administration of TNBS resulted in decreased numbers of cholinergic neurons. Cholinergic neuronal loss was attenuated by both BM-MSCs and AT-MSCs. Similar to total neuronal counts, AT-MSCs were less effective at attenuating cholinergic neuronal loss at 24 h. The unchanged proportion of cholinergic neurons may be attributed to the parallel loss of total myenteric neurons [41]. Intestinal inflammation induced by TNBS increased the total numbers and proportions of nNOS-IR nitrergic neurons. Similar changes to the neurochemical coding of enteric neurons have been observed in experimental models of colitis [80] and in Crohn’s disease patients [47, 82]. Furthermore, neurons in the central nervous system reportedly increase in nNOS expression in response to inflammatory stimuli [83, 84]. MSCs from both sources similarly attenuated neurochemical alterations in nitrergic neurons. Excessive nitric oxide has been linked to neuropathy in enteric neurons upon intestinal inflammation [85] and likewise with peripheral motor neurons [86, 87]. Thus, attenuating increases in nNOS could be partially responsible for the therapeutic action of MSCs in neuropathy.

The loss of nerve fibres in the myenteric plexus was prominent after TNBS administration in our study. TH-IR nerve fibres represent extrinsic noradrenergic sympathetic fibres in the myenteric plexus projecting to the ganglia, mucosa, and blood vessels which regulate motility and vasomotor function [77, 88, 89]. Sympathetic neurotransmitters can have pro- and anti-inflammatory effects depending on concentration [90]. Damage to sympathetic nerve fibres have been reported in Crohn’s disease patients [91]. In acute experimental colitis, sympathectomy improves outcomes but conversely has adverse effects in chronic models [91]. This suggests that sympathetic innervation may possess beneficial anti-inflammatory properties in chronic stages of intestinal inflammation. Immunoreactivity for VAChT identifies cholinergic fibres from a broad range of neurons including extrinsic vagal, intrinsic excitatory muscle motor neurons, ascending and descending interneurons, primary afferent neurons and intestinofugal afferent neurons [92]. The loss of innervation from these fibres may have repercussions in immunomodulation. Acetylcholine has been identified as a potent immunomodulator. The inflammatory reflex mediated through the efferent and afferent arms of cholinergic vagal fibres innervating the mucosa is suggested to prevent the release of pro-inflammatory mediators from macrophages via the α7 nicotinic cholinergic receptor [93, 94]. In our study, the loss of cholinergic fibres coincided with the loss of cholinergic neurons in the myenteric plexus, suggesting that most damaged fibres were of intrinsic origin. Both BM-MSC and AT-MSC treatments attenuated the loss of cholinergic and sympathetic nerve fibres to levels comparable with controls.

In the myenteric plexus, sensory extrinsic afferent fibres, as well as intrinsic afferent neurons and fibres, are immunoreactive for CGRP. Reduced CGRP immunoreactivity was observed in the myenteric plexus from inflamed animals. In ulcerative colitis, lack of CGRP expression correlates with disease activity scores and may be a useful marker of disease progression [95]. Furthermore, abrogation of CGRP signalling via neutralising antibodies or associated receptor antagonist promotes inflammation in experimental colitis [96]. Together this suggests that sensory nerve fibres may play an anti-inflammatory role. In our study, BM-MSCs and AT-MSCs attenuated the loss of CGRP immunoreactivity, although levels remained lower compared to shams. This may be reflective of chemorepulsive mechanisms inhibiting sensory fibre projections [97], which might be an endogenous mechanism to prevent pain or hypersensitivity; however, this needs to be elucidated.

The mechanisms responsible for MSC-mediated neuroprotection in the ENS are yet to be investigated. In this study, BM-MSCs were more efficacious at ameliorating neuropathy and plexitis. This correlation may suggest immunomodulatory effects are responsible for the neuroprotective properties exerted by MSCs. However, this does not explain the equality of BM-MSCs and AT-MSCs in attenuating inflammation-induced damage to nerve fibres and changes to neurochemical coding observed in our study. Both BM-MSCs and AT-MSCs release neurotrophic factors including nerve growth factor, brain derived neurotrophic factor, neurotrophin-3 and glial derived neurotrophic factor [65]. These factors have all been linked to neuroprotective effects [98–100]. Thus, the neuroprotective action of MSCs may occur via immunomodulation, directly by paracrine secretion of neurotrophic factors or synergistically through both. Furthermore, the neuroprotective efficacy of MSCs could be associated with their ability to migrate and engraft in proximity to enteric neurons.

In our study, MSCs administered by enema migrated transmurally from the mucosa to the myenteric ganglia, although most MSCs were observed in the mucosa. The low number of MSCs migrating to the muscle layers, relative to the mucosa, might be due to the short length of experiments in this study as greater numbers of MSCs migrating to the muscle layers have been observed at late stages of experimental colitis [32]. To assess the chemotactic properties of MSCs, an in vitro assay was designed to determine MSC migration toward secreted factors released by cultured cells of the myenteric plexus under simulated inflammatory conditions. Both AT-MSCs and BM-MSCs migrated towards the milieu of LPS-damaged neurons within a 72 h period. In agreement with our in vivo data, the proportion of MSCs with chemotactic affinity was low and differences were not observed between tissue sources. In other neuroinflammatory models, such as experimental autoimmune encephalomyelitis, AT-MSCs were more therapeutic than BM-MSCs due to their enhanced migratory capabilities [25]. In the rat model of intestinal inflammation, it has been reported that intraperitoneally injected AT-MSCs migrated from the peritoneum through to the epithelial layer, whereas BM-MSCs only localized to the peritoneum surface, muscular layers, and submucosa by 72 h [31]. The direction of migration suggests a high chemotactic affinity to the mucosal, or possibly submucosal, layers where tissue damage is most prominent. The dissimilarities between BM-MSC and AT-MSC migration in these studies could be explained by their differential expression of chemokine receptors [101]. In addition, the morphological analysis performed in our study may suggest that BM-MSCs, containing greater proportions of large ‘flat’ cells, may be limited in migration due to their physical size. Nonetheless, MSC migration did not appear to greatly influence the therapeutic outcomes of this study or that conducted by Castelo-Branco et al. [31]. The low affinity of MSC migration to the myenteric plexus in our study suggest that either low numbers of MSCs are required to exert therapeutic effects on myenteric neurons, or, the proximity of MSCs is irrelevant to their neuroprotective mechanism. Investigations into paracrine secretion may explain the differences observed in this study between BM-MSCs and AT-MSCs.

## Conclusions

Optimisation of MSC therapies is of critical importance for their clinical application. Identifying the ideal tissue source of MSCs for the treatment of intestinal inflammation may lead to improved therapeutic outcomes. In vitro, AT-MSCs were determined to have greater proliferation, clonogenicity, and ‘spindle’ morphology suggesting that AT-MSCs are ideal for cellular expansion. In vivo*,* both BM-MSCs and AT-MSCs ameliorated weight loss, histopathological changes, plexitis, neuropathy, changes to neuronal neurochemical coding, and loss of nerve fibres; however, BM-MSCs appeared to be more effective in the treatment of neuropathy and plexitis. These differences could not be explained by migration capacity to the myenteric plexus both in vivo and in vitro*.* Future studies should determine the role of paracrine secretion in the neuroprotective efficacy of MSCs in addition to their direct and indirect interactions with myenteric neurons. The benefits between the expansiveness of AT-MSCs and the increased efficacy of BM-MSCs to target neurological manifestation should be considered when selecting MSCs to treat intestinal inflammation.

## Abbreviations

AT-MSC: Adipose tissue-derived mesenchymal stem cells
BM-MSC: Bone marrow-derived mesenchymal stem cells
CGRP: Calcitonin gene-related peptide
CFU-f: Colony forming unit-fibroblast
ChAT: Choline acetyltransferase
ENS: Enteric nervous system
FITC: Fluorescein isothiocyanate
HLA: Human leukocyte antigen
IBD: Inflammatory bowel disease
IR: Immunoreactive
LMMP: Longitudinal muscle and myenteric plexus
MSC: Mesenchymal stem cells
nNOS: Neuronal nitric oxide synthase
PDL: Population doubling level
PGP9.5: Protein gene product 9.5
TNBS: 2,4,6-Trinitrobenzene sulfonic acid
TH: Tyrosine hydroxylase
VAChT: Vesicular acetylcholine transporter
